# Prenatal diagnosis of persistent cloaca accompanied by uterus didelphys: A case report

**DOI:** 10.4274/tjod.galenos.2020.44442

**Published:** 2021-03-12

**Authors:** Koray Görkem Saçıntı, Gizem Oruç, Erdal Şeker, Mehmet Seçkin Özışık

**Affiliations:** 1 Ankara University Faculty of Medicine, Department of Obstetrics and Gynecology, Ankara, Turkey

**Keywords:** Persistent cloaca, uterus didelphys, prenatal diagnosis, fetal pelvic mass

## Abstract

Persistent cloaca is a rare abnormality that occurs usually in females and is characterized by direct communication between the gastrointestinal, urinary, and genital structures resulting in a single perineal opening. We report a case of persistent cloaca accompanying uterus didelphys that was diagnosed antenatally with fetal ultrasonography. A gravida 3, para 2, 35-year-old women at 22 weeks of gestation was referred to our hospital with a diagnosis of moderate pyelectasis of the fetal kidneys and fetal diffuse intestinal dilation. Detailed ultrasound scan findings were reported as a small thick-walled septated cystic pelvic mass of 5.2×5.5 cm size seen at the level of the fetal pelvic region. The target sign could not be visualized, it was considered as anal atresia. In the following weeks, the patient, who was evaluated together with meconium on the uterine septum, and monitoring of the neighboring bladder and anal atresia, was diagnosed as having persistent cloaca. Ultrasound findings showed that it could be persistent cloaca accompanying uterus didelphys. The fetus postnatally manifested persistent cloaca. On the first day after vaginal delivery, pelvic ultrasound in the neonatal intensive care unit showed bilateral 2^nd^-degree hydronephrosis, presacral enlarged bowel loops, uterus didelphis, vaginal septum, direct contact between urethra and vagina, proximal end in the rectum compatible with atresia. On the second day, colostomy was performed. Her renal condition continued to be stable. She is now waiting for definitive surgery for cloaca. Persistent cloaca should be considered in any female fetus presenting with hydronephrosis and a cystic pelvic mass lesion as diagnosed by ultrasound. Prenatal diagnosis allows time for parental counseling and delivery planning at a tertiary hospital for neonatal intensive care and pediatric surgery.

## Introduction

Cloacal anomalies represent the persistence of early embryonic development in which the urinary, genital, and gastrointestinal tracts remain confluent and communicate with the exterior through a single perineal opening. It is an extremely rare disorder, the incidence being 1 in 50,000 births^(1)^. It generally affects females. There may be co-existent anomalies of the gastrointestinal tract, cardiac, renal, uterine, skeletal and limbs. The presence of a cloaca is a normal phase of early human embryologic development. Between the 4^th^ and 7^th^ weeks of gestation, the cloaca undergoes subdivision to form the hindgut and urogenital sinus. Failure of this process results in the congenital anomaly termed persistent cloaca^(2)^. Herein, we report a case of persistent cloaca accompanying uterus didelphys that was diagnosed antenatally in fetal ultrasonography.

## Case Report

**Figure 1:**
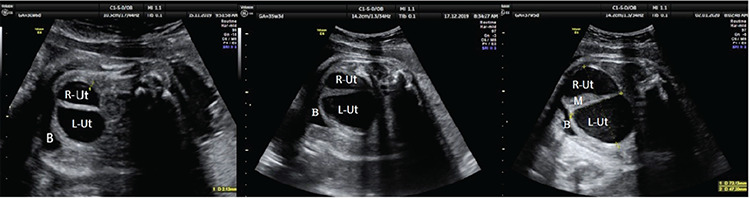
Ultrasound images show the evolution of cloaca accompanied uterus didelphys from 30, 35 and 37 weeks of gestation respectively R-Ut: The right uterine cavity, L-Ut: The left uterine cavity, B: Bladder, M: Meconium

A gravida 3, para 2, 35-year-old woman at 22 weeks of gestation was referred to our hospital with a diagnosis of moderate pyelectasis of the fetal kidneys and fetal diffuse intestinal dilation. The amniotic fluid level was normal. Detailed ultrasound scan findings were reported as a small thick-walled septated cystic pelvic mass of 5.2×5.5 cm size seen at the level of the fetal pelvic region. Except for two previous normal vaginal deliveries, her obstetric history was unremarkable. The glucose tolerance test performed at about 24 weeks during pregnancy led to the diagnosis of gestational diabetes. The target sign could not be visualized, and anal atresia was considered. In the following weeks, the patient, who was evaluated together with meconium on the uterine septum, and monitoring of the neighboring bladder and anal atresia, was diagnosed as having persistent cloaca (Figure 1). She was diagnosed as having intrahepatic cholestasis of pregnancy at 37 weeks of gestation. We decided to induce labor as determined by the Bishop score. After the initiation of misoprostol induction, in another 8 h, the patient gave birth to a live-born female infant weighing 2,520 g, a height of 47 cm, and an Apgar score of 9-10 points. The female neonate presented with an absent anal opening; she was passing stool from the vestibule. The newborn had abdominal distension, simulating a distended bladder, and rectal atresia with a single perineal opening between the labia majora. The fetus postnatally manifested as persistent cloaca. On the first day after vaginal delivery, pelvic ultrasound in the neonatal intensive care unit showed bilateral 2^nd^-degree hydronephrosis, presacral enlarged bowel loops, uterus didelphis, vaginal septum, direct contact between urethra and vagina, and the proximal end in the rectum was compatible with atresia. On the second day, colostomy and cystoscopy were performed. Her renal condition continued to be stable. She is now waiting for definitive surgery for cloaca. The patient postpartum period was uncomplicated. At the follow-up visit between 4-6 weeks, the patient was in good health and without symptoms.

## Discussion

Persistent cloaca is an uncommon malformation that generally occurs in females, with a reported incidence of 1:50,000^(1)^. It is a complex anomaly with a confluence of the rectum, vagina, and urethra into a single common channel, and can be associated with female hypospadias, duplex uteri, bladder diverticulum, and double vagina, to more complex anomalies^(3)^.

Cloacal anomalies are considered prenatally in the presence of bilateral hydronephrosis, a pelvic cystic lesion, and a poorly visualized bladder^(1)^. However, prenatal diagnoses of female urogenital anomalies are usually difficult. Accurate diagnosis and classification of cloacal anomalies during pregnancy are essential to predict perinatal morbidity and mortality. These defects are rare, manifest as varying defects, and particularly in the late stages of pregnancy, lack characteristic ultrasound signs^(4)^.

In our case, the cloacal anomaly presented as a fetal duplication cyst. The fetal pelvic septated cyst that raised clinical suspicion confirmed on the postnatal period as uterine duplication with hydrometrocolpos. Most cases of cloacal anomaly have been reported to be associated with fetal ascites^(5)^. Fetal urine drains through the fallopian tube to the peritoneal cavity and this process causes a chemical reaction that determines the tubal obstruction, hydrocolpos, and resolution of ascites, but in our case, no ascites was observed. Fetal magnetic resonance imaging (MRI) is an important diagnostic imaging adjunct to ultrasonography, particularly for the initial diagnosis of cloacal malformation^(6)^. Ultrasonography or fetal MRI can identify large cystic pelvic masses, but their origin cannot be determined in most cases.

## Conclusion

Persistent cloaca can be diagnosed prenatally and should be considered in any female fetus presenting with hydronephrosis and a large cystic lesion arising from the pelvis as assessed by ultrasound. Prenatal diagnosis allows time for parental counseling and delivery planning at a tertiary hospital for neonatal intensive care and pediatric surgery. A cystic pelvic mass in any female fetus should be evaluated for cloacal anomalies.
